# Supplementary material for: Apocynin and catalase prevent hypertension and kidney injury in Cyclosporine A-induced nephrotoxicity in rats
Source: PLoS One. 2020 Apr 16;15(4):e0231472. doi: 10.1371/journal.pone.0231472 (PMC7161975; doi:10.1371/journal.pone.0231472)
Supplement: Attachment — Submitted filename: 2nd Rebuttal.docx [file pone.0231472.s002.docx]

**Answers to reviewer’s comment**

Paper No. **PONE-D-19-26893**

Title: “**Apocynin and catalase prevent hypertension and kidney injury in Cyclosporine A induced nephrotoxicity in rats’’**

**PLOSONE**

First of all, we would like to thank the Reviewers for their comments on our manuscript. We also thank the Editor for giving his consideration to this manuscript.

**Reviewer #1:**

**NADPH oxidases are a major source of reactive oxygen species (ROS) in the kidney in normal and pathological conditions. Among NADPH oxidase isoforms, NADPH oxidase4 (Nox4) is highly expressed in the kidney and has an important role in kidney diseases.**

**Nox4 is a NAPDH oxidase expressed in the kidney and has an important role in kidney function injury. In the study, the authors only detected the mRNA expression of Nox4 in kidney tissues from control and treatment groups. mRNA expression didn’t completely reflect the protein level and the activity of the protein. How the authors can conclude that the renal injury induced by CsA was associated with the increase in NADPH oxidase activity? In addition, the authors only detect the oxidative stress makers in plasma not the kidney tissue, how to confirm that apocynin and catalase prevent the renal injury through regulating the Nox4 and oxidative stress?**

**Authors’ response:**

The aim of the study was to explore the status of expression of NADPH oxidase in cyclosporine model of kidney injury. The study emphasized that inhibition of this enzyme by administration of apocyanin and catalase will not only downregulate the expression of this enzyme but also will minimize the oxidative stress in the kidney. We showed that NADPH oxidase was indeed downregulated in the kidney by measuring NADPH oxidase mRNA by using qPCR. This downregulation of NADPH oxidase is suggested to translate into decreased protein levels of NADPH oxidase then reduction of oxidative stress biomarkers at the cellular level and in the plasma. As suggested by the Reviewer, we measured the oxidative stress biomarkers in the kidney tissue to relate the expression of NADPH oxidase mRNA with biomarker of oxidative stress. The results showed a similar trend in terms of tissue oxidative stress level as it was shown in the plasma. The levels of oxidative stress biomarkers in different groups are shown in updated Figures 4A, 4B and 4C in the updated version of the manuscript. The present study however presents the limitation that it measured NADPH oxidase mRNA and not the activity of this protein but we believe it highlighted that mRNA expression is altered in this renal injury model and in association with hypertension. Indeed, this study provides the bases for future studies to explore this gap in knowledge.

**Reviewer #2:** I recommend this manuscript for publication, however, I recommend a couple of minor edits:

**1) Abstract: Add these words in the last sentence: 'NADPH inhibition and H202 scavenging could, therefore, be a potentially important therapeutic strategy to treat CsA-induced nephrotoxicity and hypertension.'**

**Authors’ response:**

Thank you indeed for the recommending this manuscript for publication. Suggested changes regarding insertion of the above sentence has now been done.

**2) Introduction: Expand on human studies in references 6-8.**

**Authors’ response:**

Suggested changes have been done in the introduction section of this manuscript.
